# Band Structures and Transport Properties of High-Performance Half-Heusler Thermoelectric Materials by First Principles

**DOI:** 10.3390/ma11050847

**Published:** 2018-05-19

**Authors:** Teng Fang, Xinbing Zhao, Tiejun Zhu

**Affiliations:** State Key Laboratory of Silicon Materials, School of Materials Science and Engineering, Zhejiang University, Hangzhou 310027, China; fangteng_123@163.com (T.F.); zhaoxb@zju.edu.cn (X.Z.)

**Keywords:** thermoelectric, half-Heusler, first-principles, band structure, transport properties

## Abstract

Half-Heusler (HH) compounds, with a valence electron count of 8 or 18, have gained popularity as promising high-temperature thermoelectric (TE) materials due to their excellent electrical properties, robust mechanical capabilities, and good high-temperature thermal stability. With the help of first-principles calculations, great progress has been made in half-Heusler thermoelectric materials. In this review, we summarize some representative theoretical work on band structures and transport properties of HH compounds. We introduce how basic band-structure calculations are used to investigate the atomic disorder in n-type *M*NiSb (*M* = Ti, Zr, Hf) compounds and guide the band engineering to enhance TE performance in p-type Fe*R*Sb (*R* = V, Nb) based systems. The calculations on electrical transport properties, especially the scattering time, and lattice thermal conductivities are also demonstrated. The outlook for future research directions of first-principles calculations on HH TE materials is also discussed.

## 1. Introduction

Thermoelectric (TE) materials have been attracting intensive attention due to their ability of directly converting heat into electricity, and can play an important role in improving energy efficiency. These environmentally friendly and extremely reliable solid state devices have no moving parts, hazardous liquids, or greenhouse emissions. A key challenge is to improve the TE conversion efficiency in order to expand the role of TE materials and, in turn, enable new practical applications. The efficiency of TE materials depends on the dimensionless figure of merit *zT* = *α*^2^*σT*/(*κ*_e_ + *κ*_L_), where *α*, *σ*, *T*, *κ*_e_, and *κ*_L_ are the Seebeck coefficient, electrical conductivity, absolute temperature, and electronic and lattice contributions to the total thermal conductivity *κ*, respectively [1].

Among those quantities, *α*, *σ*, and *κ*_e_ are related to the electronic structure of the material. The three parameters are intercorrelated and cannot be optimized independently. For instance, increasing *α* is usually accompanied by a decreasing *σ*; an increase in *σ* concomitantly leads to an increase in *κ*_e_ via the Wiedemann-Franz law *κ*_e_ = *LσT* (*L* is the Lorenz number). One possible way to optimize *zT* is to maximize the power factor (*α*^2^*σ*), which can be achieved by the band engineering [2]. The band structure is one of the basic characteristics of materials, as well as the vital tool in understanding, optimizing, and even designing novel functional materials [3]. Once the electronic structure calculation is done, the electrical transport properties can be effectively tuned according to the band structure–related parameters. Additionally, new TE materials with high power factors can also be screened using the band structures combined with Boltzmann transport theory.

For TE materials, the main characters of the band are reflected by the effective mass [4]. In degenerate semiconductors, *α* under parabolic band approximation is a function of the density of states (DOS) effective mass m^*^ [5]. According to the formula m^*^ = *N*_v_^2/3^*m*_b_^*^ (*N*_v_ is the band degeneracy and *m*_b_^*^ is the band effective mass), increasing both *N*_v_ and *m*_b_^*^ contributes to a enhanced m^*^ and, consequently, *α* [6]. However, a high *m*_b_^*^ always leads to a low carrier mobility *μ* due to *μ* ∝ 1/*m*_b_^*^. It has been proved that increasing *N*_v_ is beneficial to large m^*^ without deterioration of *μ*, and is an efficient strategy to improve TE performance for many materials [7,8,9,10].

The lattice thermal conductivity *κ*_L_, related to its phonon vibration, is more or less independent of the electronic transport properties. However, defects that reduce *κ*_L_, such as forming solid solutions or making composite structures, are likely to reduce *μ* [4]. Nevertheless, the differences between the mean free paths of phonons and electrons open the window for nanostructuring technology at a length scale that scatters phonons, but not electrons [11]. Alternatively, seeking promising TE materials with low thermal conductivity attracts much attention [12,13,14]. Only phonon-phonon Umklapp scattering is considered, *κ*_L_ ∝ *MV*^1/3^*θ*_D_^3^/*γ*^2^ (*M* is the average mass per atom, *V* is the average atomic volume, *θ*_D_ is the Debye temperature and *γ* is the Grüneisen parameter) [15]. Accordingly, low *M*, *V*, *θ*_D_, and high *γ* contribute a low *κ*_L_. Starting with this viewpoint, many good TE materials with intrinsically low *κ*_L_ have been reported [16,17,18].

**Figure 1:**
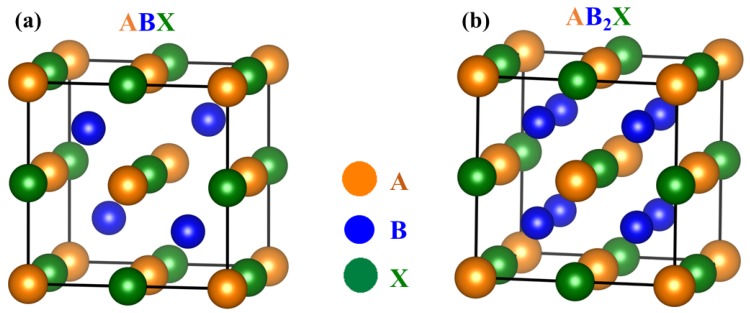
Crystal structure of (**a**) half-Heusler ABX and (**b**) Heusler AB_2_X compounds.

Half-Heusler (HH) compounds are ternary intermetallics with a general formula ABX, where A and B are usually transition metals and X is a main group element [19], as shown in Figure 1a. A typical HH compound takes the form of the MgAgAs structure type with space group F$\bar{4}$3m. It consists of three filled interpenetrating face-centered cubic (fcc) sublattices and one vacant fcc sublattice [20]. The Heusler structure with formula AB_2_X is obtained by filling the vacant sublattice with B atoms, as shown in Figure 1b. The most electronegative element X and the most electropositive element A usually form the NaCl sublattice with octahedral coordination, leaving the all-etrahedral site to the intermediate electronegative element B [21]. The properties of HH compounds depend strongly on the valence electron count (VEC) of the constituent elements. HH compounds, with VEC = 8 or 18, are usually semiconductors with excellent potential as TE materials.

As is known, the fabrication of single-phase HH compounds is difficult, which is mainly due to the distinct differences in the specific gravity and melting point of the constituent elements [22]. Note that even if the samples are single-phase, atomic antisite disorders in n-type *M*NiSn (*M* = Ti, Zr, Hf) HH compounds have profound effects on TE properties [23,24]. In this regard, first-principles calculations have played an important role in providing a theoretical basis for observed experimental phenomena. The earliest report about structure disorder was Zr/Sn antisite defect in ZrNiSn [23]. The change in the content of the Zr/Sn disorder resulted in a significant change in the band gap and TE properties [25]. Recently, combined with experimental studies, band structure calculations showed that the effects on the band gap and TE properties are mainly due to Ni/vacancy disorder [26,27], instead of Zr/Sn antisite. In addition, band structure calculations enable the design of HH-based TE materials through band engineering. Recently, p-type Fe*R*Sb (*R* = V, Nb) HH compounds are expected to exhibit attractive TE performance [28]. A high *zT* has been obtained in these heavy-band semiconductors via a band engineering approach [29].

It is also worth mentioning that first-principles calculations have provided the guideline for developing new promising HH-based TE materials. Up to now, experimental investigations on HH compounds have mainly focused on n-type *M*NiSn [30,31,32,33,34]. There is only a very small fraction of studies on other HH systems [35,36]. Actually, more than 100 HH compounds can be found in the Inorganic Crystal Structure Database (ICSD) [37]. Even using the criterion of VEC = 18, there are still more than 30 HH compounds left [38]. There is no denying that the process of discovering new TE materials is limited by the high cost and the time-consuming procedures of experiments. The situation is more severe for HH compounds due to the difficulty in preparing single-phase samples. Recently, ab initio-based calculations and high-throughput material screening based on some models bring up an avenue of material discovery. Beneficial from the simple crystal structures of HH compounds, the computer-aided material design is manipulable, computationally.

In this paper, we focus on the recent significant progress in theoretical investigation on HH-based TE materials. In Section 2, we describe how basic band-structure calculations are used to understand the transport properties of HH compounds and provide the guideline for developing new promising HH compounds. In Section 3, we introduce some different treatments on relaxation time in calculating electronic transport properties of HH compounds. In Section 4, we present some high-throughput calculations on lattice thermal conductivities of HH compounds.

## 2. Manipulating the Band Structures of HH Compounds

Band structures, which reside in reciprocal space due to the periodicity of the lattice, directly determine the electrical transport properties of materials. The accurate band structures are beneficial for understanding the underlying transport physics and designing new TE materials. Therefore, it is crucial to establish the connections between the electrical transport properties in real space and the band structures in reciprocal space. The calculation of band structures became accessible with the development of density functional theory (DFT) [39,40], which is usually used in the field of TE materials.

### 2.1. Band Structures and Atomic Disorders in N-Type MNiSn

In 1995, Öğüt and Rabe [41] calculated the band structures of *M*NiSn alloys using DFT methods. The indirect band gaps of ~0.5 eV were found between the *Γ* and *X* points, which are larger than experimental ones (0.12, 0.19 and 0.22 eV for TiNiSn, ZrNiSn, and HfNiSn, respectively) [24]. Öğüt and Rabe ascribed this difference to the Zr/Sn antisite disorder, which was firstly reported experimentally in the ZrNiSn by Aleiv et al. [23]. The calculations for antisite defects, which were carried out using virtual crystal approximation (VCA), showed that the band gap would be reduced by increasing the content of Zr/Sn antisite defects until going to zero with ≈15% Zr/Sn disorder [41]. Qiu et al. also reported the decreased band gap of ZrNiSn with the increase of antisite defects concentrations using direct ab initio calculations [25]. By changing the degree of antisite defects with different annealing periods, the TE properties can also be influenced greatly.

**Figure 2:**
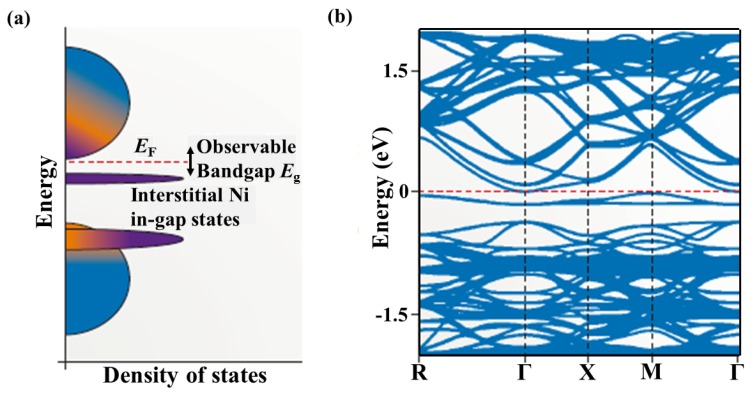
In-gap state formation via *d* orbitals from interstitial Ni. (**a**) Schematic diagram of the density of states in *M*NiSn showing the in-gap Ni states (purple) resulting from intrinsic defects. These extra states in the band gap lead to a smaller observed optical band gap. (**b**) Calculated band structure showing the band within the energy gap near the conduction band edge. Reproduced with permission [46]. Copyright 2016, Nature Publishing Group.

However, Larson et al. suggested that antisite defects of Ni atoms occupying the vacant sites also exist in *M*NiSn compounds [42]. An analysis of the energetics of antisite defects showed that the Ni/vacancy disorder would cause a smaller energy increase above the perfect lattice structure, compared to the Zr/Sn antisite disorder. The N-related disorder was also proved experimentally by the in-gap electronic states close to the Fermi energy due to the existence of two types of Ni atoms with different electron occupancies [43,44]. Xie et al. found that there was no evidence to support the existence of Zr/Sn antisite defects in the single-phase ZrNiSn prepared by levitation melting and not subjecting to annealing. However, the excess charge density in Fourier map and Rietveld refinement analysis indicated the existence of a fractional occupancy of Ni on the vacant 4*b* site [26]. Douglas et al. [45] and Do et al. [27] suggested that excess Ni atoms in *M*NiSn compounds tend to stay close to each other to form nanoclusters and showed the in-gap states from interstitial Ni near the conduction band edge in the calculated band structures (Figure 2). The band gap of TiNi_1+x_Sn is reduced from 0.45 to 0.12 eV due to the Ni interstitial, which is consistent with the experimental gap of 0.12 eV for TiNiSn [24]. Recently, Zeier et al. ascribed the in-gap band to that Ni vacancies or excess Ni should be largely electron neutral (effectively Ni^0^ by assigning bonding (NiSn)^4−^ orbitals to Sn as Sn^4−^). In this case, non-stoichiometry in the form of ZrNi_1+x_Sn may be expected to not move the Fermi level outside the band gap [46].

Here we have to point out that the band structure calculations by Douglas et al. [45] and Do et al. [27] were done using the GGA-PBE density functional, which is known to underestimate the band gaps of semiconductors. For example, the calculated band gaps of TiNiSn with the GGA-PBE density functional, HSE06 density functional and the *G*W_0_ method are 0.45, 0.62, and 0.75 eV, respectively [47]. The differences are mainly due to that the observed relative positions of the *d* levels in the transition metal atoms vary among the different methods. However, considering that calculations using the *G*W_0_ method are very computationally expensive for defect calculations due to the large supercells, more precise and computationally-tractable methods should be applied to the *M*Ni_1+x_Sn systems.

### 2.2. Performance Optimization of P-Type Heavy-Band HH Using Band Engineering

Fe*R*Sb (*R* = V, Nb)-based HH compounds, with abundantly available constituent elements, have attracted signfiicant attention due to a high Seebeck coefficient (approximately –200 μV K^−1^ at 300 K) and a large power factor (4.5 × 10^−3^ W m^−1^ K^−2^ at 300 K) [48]. However, due to a relatively high lattice thermal conductivity (10 W m^−1^ K^−1^ at 300 K), earlier studies have been focused on improving the TE performance of n-type Fe*R*Sb by alloying [49,50] or nano-structuring [51], but only a marginal improvement in *zT* (≈0.33 at 650 K) was obtained.

**Figure 3:**
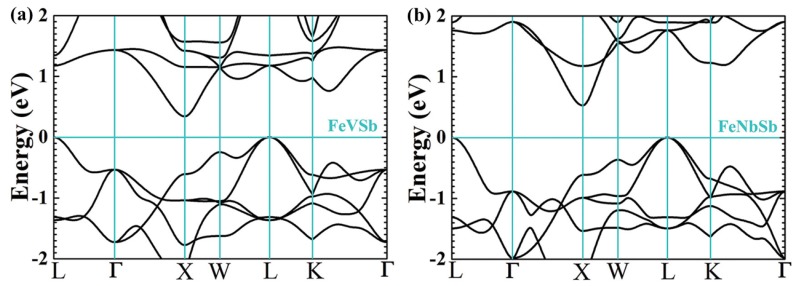
Electronic band structure for (**a**) FeVSb and (**b**) FeNbSb. Reproduced with permission [6]. Copyright 2015, Wiley.

Recently, band structure calculations showed that the characteristic of the valence band shows great disparity from that of conduction band in Fe*R*Sb (Figure 3). The conduction band minimum of Fe*R*Sb locates at point *X* with a band degeneracy of *N*_v_ = 3. In comparison, the valence band maximum of Fe*R*Sb lies in point *L* with a higher band degeneracy of *N*_v_ = 8 [52,53], which is beneficial for TE performance as a large DOS effective mass m^*^ is desired for good TE materials [2,7]. According to the formula m^*^ = *N*_v_^2/3^*m*_b_^*^ and *µ* = 1/*m*_b_^*5/2^, large *N*_v_ is beneficial for large m^*^ without deterioration of *µ*. Therefore, increasing *N*_v_ is an effective way to improve TE performance of a material without deteriorated side effects. TE properties of p-type Ti-doped FeV_0.6_Nb_0.4_Sb solid solutions were first investigated [28]. Combined with the high *N*_v_ of 8 and heavy *m*_b_^*^ of 2.5 *m*_e_, a high m^*^ of 10 *m*_e_ was obtained in the p–type Fe(V_0.6_Nb_0.4_)_1−x_Ti_x_Sb compounds, which resulted in a high Seebeck coefficient. Although the heavy *m*_b_^*^ led to a low *µ*, the low deformation potential and alloy scattering potential were both beneficial for a reasonably high mobility in this system. Therefore, a high power factor of about 3 × 10^−3^ W m^−1^ K^−2^ was available at 900 K for Fe(V_0.6_Nb_0.4_)_0.8_Ti_0.2_Sb. Mainly due to the high power factor, in addition to the relatively low lattice thermal conductivity among the Fe*R*Sb system, a high *zT* of ≈0.8 at 900 K was achieved.

**Figure 4:**
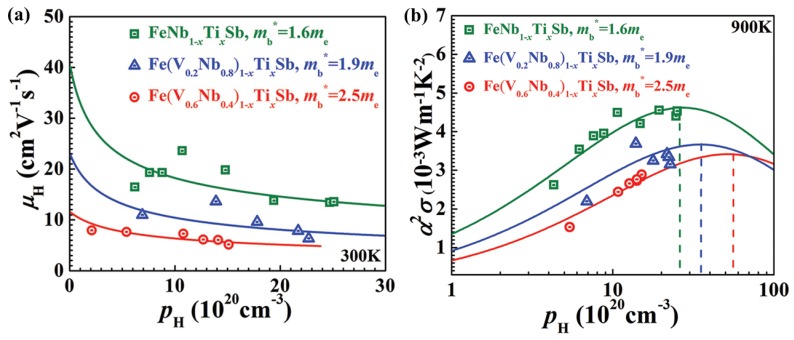
Hall carrier concentration dependence of (**a**) carrier mobility and (**b**) power factor for p-type Ti doped Fe(V_1−y_Nb_y_)_1−x_Ti_x_Sb HH compounds. Reproduced with permission [29]. Copyright 2014, Royal Society of Chemistry.

It is obvious that the heavy *m*_b_^*^ leads to a low *µ* in Fe(V_0.6_Nb_0.4_)_1−x_Ti_x_Sb. Based on the band structures, the *m*_b_^*^ of 0.16 *m*_e_ for p-type FeNbSb is lower than that of 0.25 *m*_e_ for p-type FeVSb, indicating that increasing Nb content may lead to a lower *m*_b_^*^ and, hence, higher *µ* (Figure 4a). Moreover, the *m*_b_^*^ decrease can lower optimal carrier concentration [54]. The solubility limit of Ti in Fe(V_0.6_Nb_0.4_)Sb was about 20%. The optimized power factor may be realized within the solubility limit of Ti by decreasing the optimal carrier concentration (Figure 4b). The band gap of 0.54 eV for FeNbSb is also larger than that of 0.34 eV for FeVSb, meaning that higher Nb content in Fe(V_0.6_Nb_0.4_)_1−x_Ti_x_Sb will broaden the band gap and consequently increases the temperature at which bipolar diffusion begins to diminish TE performance. The enhanced carrier mobility and reduced optimal carrier concentration result in the optimal power factor. The power factor of p-type FeNb_0.8_Ti_0.2_Sb was about 4.5× 10^−3^ W m^−1^ K^−2^ at 1100 K, ~50% higher than the Fe(V_0.6_Nb_0.4_)_1−x_Ti_x_Sb solid solutions. A higher *zT* value of 1.1 at 1100 K was achieved for FeNb_0.8_Ti_0.2_Sb due to the enhanced power factor [29].

**Figure 5:**
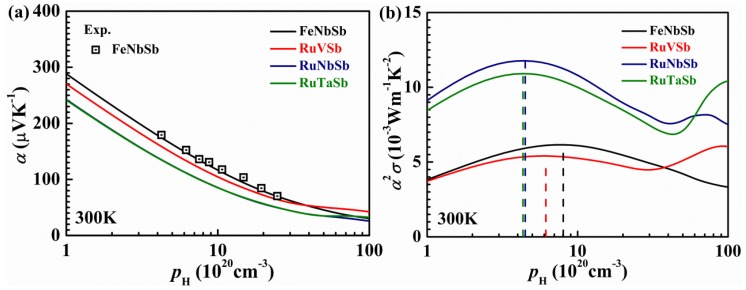
The calculated carrier concentration dependence of (**a**) Seebeck coefficient and (**b**) power factor for Ru*M*Sb (*M* = V, Nb, Ta) samples at 300 K [58]. Reproduced by permission of the PCCP Owner Societies.

Although the valence band of FeNbSb is sharper than that of FeVSb, the m^*^ of 6.9 *m*_e_ for FeNb_1−x_Ti_x_Sb is also higher than that of 0.3 *m*_e_ and 1.3 *m*_e_ for conventional PbTe-based and Bi_2_Te_3_-based materials, respectively [54,55]. The large *m*_b_^*^ in these Fe-containing p-type TE materials is due to the spatially localized nature of transition metal 3*d* orbitals [56]. It has been reported that p-type skutterudites containing 4*d* transition metal, such as Ru, possess low *m*_b_^*^ at the valence band [57], which is beneficial for high carrier mobility. Therefore, Ru-based HH alloys may also be promising TE materials with low band effective mass. The calculated band structures showed that the valence bands of Ru-based HH alloys are lighter than that of Fe-based compounds (Figure 5a) [58]. The power factors of p-type RuNbSb and RuTaSb are about 100% higher than that of p-type FeNbSb due to the lower *m*_b_^*^ and hence higher *µ* (Figure 5b). Moreover, the lattice thermal conductivities of RuNbSb and RuTaSb are also lower than FeNbSb, exhibiting high potential for high-temperature TE power generation.

**Figure 6:**
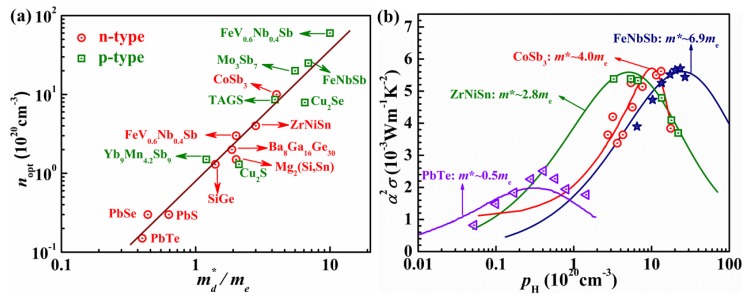
(**a**) The optimal carrier concentration versus the density of state effective mass for TE materials. The solid line is a guide for eyes; (**b**) carrier concentration dependence of the power factor for the typical light-band PbTe, and the heavy-band system: n-type ZrNiSn, n-type filled CoSb_3_ and p-type FeNbSb near 800 K. Reproduced from [59].

Actually, filled skuterudites and HH compounds, usually containing transition metal elements with localized *d* states, are heavy-band materials due to the flat valence band maximum or conduction band minimum [38,56]. Typically, the m^*^ of these heavy-band materials are in the range of 2 *m*_e_–10 *m*_e_ [59] (Figure 6a). Accordingly, higher carrier concentrations, which demands for higher contents of dopants, are necessary to optimize the power factors. Even though these heavy-band TE materials have low *µ*, their optimal power factors are 2–3 times higher than that the state-of-the-art light-band PbTe (Figure 6b). Combined with the strong point-defect phonon scattering due to a high content of dopant, high power factors make these heavy-band TEs promising for power generation. Therefore, the tradeoff between the band effective mass and carrier mobility is crucial to the TE performance of these heavy-band semiconductors.

## 3. Electronic Transport Properties of HH Compounds

The Boltzmann transport theory is usually an appropriate description for electrical transport in TE materials, due to their relatively high operating temperatures and macroscopic sizes of the samples [60]. The electronic transport coefficients of TE materials (*α*, *σ*, and *κ*_e_) are ultimately determined by the transport distribution function [61], Σ(*E*) = *v*^2^*τg* (where *E* is the energy, *v*, *τ*, and *g* are the group velocity, the relaxation time, and the density of states, respectively). Currently, the greatest challenge for computations is to capture the relaxation time *τ*, which is affected by many scattering mechanisms and difficult to calculate accurately [62]. The energy dependence of *τ* impedes the direct evaluation for large sets of TE materials. Thus, several approximations for *τ* have been proposed for quantifying the TE performance of materials.

### 3.1. Constant Relaxation Time Approximation

Constant relaxation time (CRT) approximation is the most common approach in solving the Boltzmann transport equations [63]. In this approximation, *τ* is assumed to be energy independent, leading to a computationally manipulable form of the equations for electronic transport coefficients. The calculations of electronic transport properties within the CRT approximation are most commonly performed using the BoltzTraP code [64], which combines electronic structure calculations and the Boltzmann transport theory.

**Figure 7:**
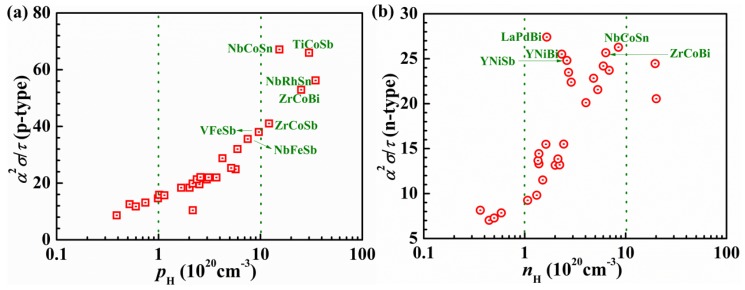
Maximum power factors vs the corresponding carrier concentrations for (**a**) p-type and (**b**) n-type cases. Reproduced with permission [38]. Copyright 2008, Wiley.

In one of the earliest applications of BoltzTraP to screen for TE materials, the electronic structures and electronic transport properties of 36 HH compounds were systematically investigated [38]. Calculated band structures showed that the band gaps of these HH compounds depend sensitively on transition metals, whose *d*-states are the dominant electronic states around band gaps. Therefore, changing elements at X positions does not substantially affect the gap value. Additionally, there is no strong correlation between the band gap and the corresponding elemental electronegativity differences. The maximum power factor and the corresponding optimal n-type and p-type doping levels were also determined. The calculated optimal doping levels and the corresponding Seebeck coefficients exhibited reasonable agreement with experiments for five previously-studied HH compounds. Figure 7 shows the relationship between the maximum power factor and the corresponding optimal carrier concentration for both p- and n-type HH compounds. For p-type materials, the HH compounds containing Co, Fe, and Rh usually possess relatively high power factors (Figure 7a). The corresponding carrier concentrations are over 10^21^ cm^−3^. Based on the estimated n-type power factors, IIIB-(Ni, Pd) and some Co-containing HH compounds show reasonable n-type performance and their carrier concentrations fall in the range of 10^20^–10^21^ cm^−3^ (Figure 7b). Recently, Fu et al. have proved that p-type FeNb(V)Sb are promising HH-based TE materials, verifying the CRT approximation in the study of HH compounds.

Given that *τ* is dependent on energy, treating *τ* as an energy-independent constant is a severe limitation of CRT approximation. However, for the compounds with the same crystal structures and similar chemical compositions, CRT approximation is expected to provide a systematic evaluation about their relative performance.

### 3.2. Constant Mean Free Path Approximation

An alternative approach is the constant mean free path (*λ*) approximation (CMFP) [65]. Within this approximation, *τ* becomes energy-dependent (*v*(*E*)*τ*(*E*) = *λ*) and *λ* is constant for different material systems. To ensure the same *λ*, it is necessary to assume that materials are nanostructured, with *λ* limited by the grain size. Furthermore, the grain size needs to be smaller than the smallest *λ* in the considered materials. Although this approach is not suitable for materials with intrinsically long *λ*, it may be able to evaluate the electrical transport properties for systems with naturally low *µ* and, hence, *λ*.

Carrete et al. used the CMFP approximation to investigate 75 nanograined HH compounds, screened from 79,057 HH compounds included in the AFLOWLIB.org consortium repository [66]. The selection criteria were including formation enthalpy, phonon dispersion, ternary phase diagrams, and spin-polarized calculations. Then ab initio modeling of *zT* was performed for 75 nanograined compounds. The calculated *zT* values of many HH compounds are higher than those of nanograined IV and III-V semiconductors. Especially remarkable are the values of *zT* in excess of 2 achieved for about 15% of them at *T* > 600 K. Although the *zT* values of best n-type doped compounds are comparable with those of the best p-type doped ones, the general trend is that a typical p-type doped HH shows a higher *zT* than a typical n-type doped HH. This phenomenon is mainly due to the fact that for 65% of compounds the effective mass of holes (*m*_h_^*^) is higher than that of electrons (*m*_e_^*^) according to their band structure calculations. Using the Spearman rank correlation coefficient Σ [67], power factor is found to be a better predictor of *zT* than lattice thermal conductivity. At both room and high temperatures, the power factor depends most markedly on the band effective mass and band gap. Carrete et al. also provided simple rules to determine if nanograined HH compounds are likely to be good TE materials through machine learning techniques. In this work, they only considered a few elements’ properties, such as atomic numbers and masses, positions in the periodic table, atomic radii, Pauling electronegativities [68], and Pettifor’s chemical scales [69]. Five promising HH compounds were ultimately identified for room temperature and high-temperature applications. BiBaK, AuAlHf, and CoBiZr are the best candidates at both temperatures. Unfortunately, the accuracy of this approach cannot be verified since the predicted candidates have not been investigated experimentally.

### 3.3. Calculations of Relaxation Times

Despite that CMFP approximation treats *τ* as energy-dependent, the small grain sizes (the order of several nanometers) impede its widespread application. Therefore, CRT approximation becomes the most used approach to calculate the electronic transport properties of TE materials even with some uncertainties. In this approximation, *α* is independent of *τ*, whereas *σ* and *κ*_e_ are both directly proportional to *τ*. An actual value of *τ* is still needed to calculate the power factor and *zT*. The simplified *zT* (*zT*_e_ = *α*^2^*σT*/*κ*_e_) has been used to identify candidate TE materials [70]. This method does not require knowledge of the value of *τ*, but *zT*_e_ is always greater than *zT*, since the lattice thermal conductivity *κ*_L_ is treated as zero. Alternatively, an approximate value *τ* from the experimental electrical conductivity can also be used to compute *zT*, assuming that *τ* is direction independent and a constant at a certain specific temperature and carrier concentration [71].

**Figure 8:**
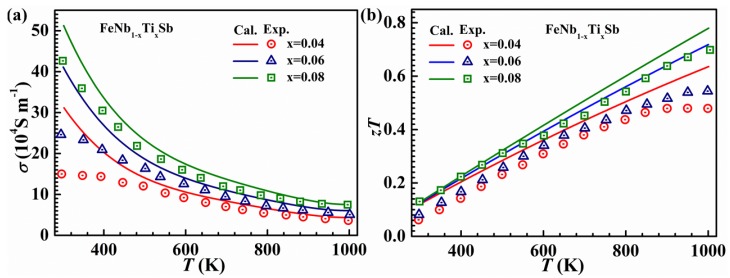
The temperature dependence of (**a**) electrical conductivity and (**b**) *zT* for FeNb_1−x_Ti_x_Sb alloys (x = 0.04, 0.06 and 0.08). The solid lines are the calculated results and the dots are measured data [29]. Reproduced from [72].

Recently, Hong et al. reported that deformation potential (DP) theory combined with the effective mass approximation is accurate to calculate *τ* of FeNbSb HH compounds [72]. In this method, *τ* ~ *c_ii_/*(*m*_b_^*3/2^ *Ξ*^2^) (where *c_ii_* and *Ξ* are elastic constant and DP constant, respectively), in which *m*_b_^*^ is very important to calculate *τ*. In their work, the *m*_b_^*^ at all *k* points in the first Brillouin zone has been calculated, and then the average of the effective masses at all specific energies can be obtained. The calculated *m*_b_^*^ near the VBM is ~1.87 *m*_e_, a little higher than the experimental value of 1.6 *m*_e_ [29]. The calculated values of *σ* are a little higher than the experimental values of FeNb_1−x_Ti_x_Sb (x = 0.04, 0.06 and 0.08) at low temperatures (Figure 8a). This discrepancy can be partially ascribed to other scattering processes (grain boundary scattering and so on), which cannot be ignored at low temperatures. A good agreement between the calculated and experimental *σ* at high temperatures indicates that the carrier scattering processes can be neglected. Due to the overestimation of *σ*, the calculated *zT* values are larger than the experimental ones at low temperatures (Figure 8b). However, the difference in *zT* between the calculation and experiment is even greater at high temperatures, which may be due to the underestimation of *κ*_L_. The calculated *κ*_L_ is smaller than the measured one at high temperature, which results in the contribution from optical phonon to thermal conductivity being neglected in the calculation. As is known, the TE properties depend substantially on the microstructures and associated defects. There are many carrier and phonon scattering processes in p–type FeNbSb-based samples [73]. Only the electron-phonon interaction for the carrier and phonon-phonon Umklapp and point-defect scatter for the phonon were considered in their scheme. Substantial uncertainties may arise and, predictably, the electrical conductivity and thermal conductivity show deviations from the measured results. However, accurate descriptions for a variety of scattering processes is difficult. Considering the approximations used and the uncertainties in experimental data, the agreement between calculated and experimental results is reasonable and acceptable.

## 4. Lattice Thermal Conductivities of HH Compounds

Above the Debye temperature, the lattice thermal conductivity is mainly dominated by phonon-phonon Umklapp scattering. Actually, other phonon-scattering processes, including point defect scattering and grain boundary scattering, also contribute to the lattice thermal conductivity. The most widely used model for predicting *κ*_L_ is the Debye-Callaway model [74]. In this model, Grüneisen parameter (*γ*), which describes the strength of lattice anharmonicity, is set to be a constant. The predicted *κ*_L_ using this model showed fairly good agreement with experimental values at room temperature [75].

Considering the existence of lattice anharmonicity, third-order anharmonic force constants are computationally necessary to determine *τ*. Using second- and third-order interatomic force constants as inputs, *κ*_L_ can be calculated via solving the phonon Boltzmann transport equation. Using this fully ab initio approach, Andrea et al. reported the *κ*_L_ of 15.4, 13.3, and 15.8 W m^−1^ K^−1^ at 300 K for TiNiSn, ZrNiSn and HfNiSn, respectively [76]. The calculated values of *κ*_L_ were different from the experimental ones, which may be due to the different defects within the samples. Katre et al. revealed that Ni/vacancy antisites are the dominant defects affecting thermal transport in ZrNiSn [77]. The calculated temperature and concentration dependence of thermal conductivities were in quantitative agreement with the published experimental results.

Even though the fully ab initio approach is accurate, it cannot be implemented in high-throughput studies due to the computational requirements of third-order force constants. In this case, some semi-empirical models for high-throughput calculations are more simplistic and computationally tractable.

Recently, Carrete et al. predicted the *κ*_L_ of 75 thermodynamically-stable ordered HH alloys based on a combination of machine learning algorithms and automatic ab initio calculations [78]. Three approaches were used in the calculations of *κ*_L_. The first method is based on the empirical observation that the force constants show a high degree of transferability between compounds sharing a crystal structure [79]. They calculated approximated *κ*_transf_ with anharmonic force constants from Mg_2_Si, since it shares the HH lattice with sites A and B occupied by Mg atoms. For cross-validation, the anharmonic force constants and *κ*_ω_ of 32 HH systems were also computed using the fully ab initio approach. The second proposed approach is calculating *κ*_forest_ via random-forest regression algorithm by leveraging the fully calculated *κ*_ω_ of 32 HH compounds as a training set. The third method presents a new machine-learning descriptor of *κ*_ω_ that integrates only the crucial pieces of the anharmonic properties of the solid. They calculated *κ*_anh_ with four exact anharmonic force constants and a linear model for the rest. The lattice thermal conductivities calculated with different methods for TiNiSn, ZrNiSn and HfNiSn are shown in Table 1. It is clear that *κ*_forest_ and *κ*_anh_ quantitatively agree well with *κ*_ω_ and *κ*_L_ calculated using the fully ab initio approach, while *κ*_transf_ shows an obvious discrepancy with *κ*_ω_ and *κ*_L_.

**Figure 9:**
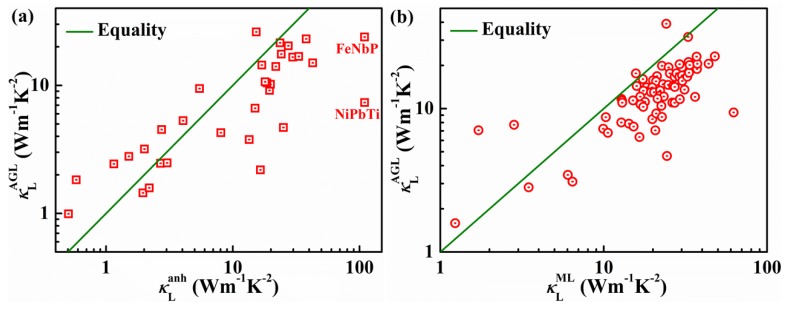
Thermal conductivities of HH semiconductors at 300 K compared to (**a**) full anharmonic phonon ab initio parametrization and (**b**) machine learning algorithm predictions from [78]. The green line represents that the value of the *x*-axis is equal to that of the *y*-axis. Reproduced with permission [80]. Copyright 2014, American Physical Society.

Subsequently, Toher et al. also calculated the *κ*_L_^AGL^ of 75 thermodynamically-stable ordered HH alloys using a quasiharmonic Debye model, which includes anharmonic contributions to a certain extent [80]. The difference between *κ*_L_^AGL^ and *κ*_L_^anh^ (*κ*_ω_ in Table 1) of 32 HH compounds is acceptable except the values of FeNbP and NiPbTe (Figure 9a), and the corresponding Spearman correlation is 0.810. However, the values of *κ*_L_^AGL^ do not agree with those of *κ*_L_^ML^ (*κ*_anh_ in Table 1), as shown in Figure 9b. The corresponding Spearman correlation is 0.706. Additionally, the predicted values of FeVSb and CoZrSb show deviations of almost an order of magnitude from the measured room-temperature values. Typically, the *κ*_L_^AGL^ of TiNiSn, ZrNiSn, and HfNiSn are 10.7, 10.22, and 12.97 W m^−1^ K^−1^, respectively. These values are much lower than *κ*_ω_ and *κ*_L_ in Table 1. Therefore, the accuracy of high-throughput calculations varies with the different models used. Although the fully ab initio approach to calculating lattice thermal conductivity is more accurate, the calculation of third-order force constants is computationally costly. Moreover, the accuracy of calculations using some simpler models is comparable to that of ab initio calculations. Therefore, high-throughput calculations can guide the search on new TE materials with low lattice thermal conductivity to some extent.

## 5. Conclusions and Outlook

HH compounds, containing hundreds of semiconductors, are a vital group of materials for high temperature thermoelectrics. Recent progress in TE performance optimization achieved high *zT* values above 1000 K for both n-type *M*NiSb (*M* = Ti, Zr, Hf) and p-type Fe*R*Sb (*R* = V, Nb) HH compounds. Indeed, the first-principles calculations have helped with the understanding of experimental results and the rationalization of experimental approaches and speeding up the new investigation of TE materials. Basic band structure calculations are beneficial for the deep understanding of intrinsic defects in n-type *M*NiSb compounds and the rational design and development of p-type Fe*R*Sb HH alloys. By density functional theory, combined with Boltzmann transport theory, electronic transport properties of some types of HH compounds have been calculated to screen new promising TE materials with excellent electrical properties. Lattice thermal conductivities of HH compounds have also been investigated via fully ab initio or high-throughput calculations based on some semi-empirical models.

Although first-principles calculations have been widely used in the research of HH-based TE materials, the limitations of first-principles cannot be ignored. There are some approximations and semi-empirical models in the calculations. Therefore, the predicted transport properties of individual materials will have some intrinsic uncertainty. However, for a class of materials with the similar structures, the calculated results are expected to be comparable with each other and could provide a systematic evaluation about their transport properties. Certainly, assessing prediction accuracy from experimental work is also needed. It is a significant challenge to obtain relevant experimental results in a timely manner for each candidate identified in a first-principles calculation. Typically, the intrinsic defects may be present in many cases and the solubility limit of extrinsic dopants need to be identified to control the carrier concentration. Therefore, assessing the dopability of new TE materials is imperative [81,82].

Due to the very large influence of the valence electron count on the electronic structure and physical properties of HH compounds, the 18-electron rule is widely used in the exploration of new promising HH phases [83]. Recently, some non-18 electron compounds have been reported to show good TE performance [84]. Typically, Zeier et al. reported that nominal 19-electron NbCoSb actually contains a HH phase with the composition Nb_0.84_CoSb using synchrotron X-ray diffraction and DFT calculations [85]. After that single-phased HH compounds Nb_0.8+*δ*_CoSb (0 ≤ *δ* < 0.05), with a remarkable enhancement on TE performance, have been successfully synthesized by levitation melting [86]. Recently, Anand et al. proposed a valence-balanced rule to understand the ground state stability of HH compounds. In other words, their ground state structures always have a common net valence of 0, regardless of stoichiometry and nominal electron count (8, 18, or 19) [87]. Using this rule, 16 nominal 19-electron HH compounds, which have not been reported previously, were predicted. The newly-predicted off-stoichiometric HH compound Ti_0.75+x_PtSb was successfully synthesized and confirmed using X-ray studies. The work on nominal 19-electron compounds opens a new avenue to search for potential HH-based TE materials theorically and experimentally. Also worth noting is that Tang et al. reported a narrow solubility range on the Ti-Ni-Sn phase diagram primarily in the range of TiNi_1+x_Sn (0 ≤ x ≤ 0.06) at 1223 K using phase boundary mapping, which explains the large discrepancy of the literature data on the thermoelectric properties of TiNiSn within a unified phase diagram framework [88]. This work also suggests a direction of research on HH thermoelectric materials by the interplay of theory and experiment.

## Figures and Tables

**Table 1:** The lattice thermal conductivities calculated with different methods for TiNiSn, ZrNiSn, and HfNiSn. Unit: W m^−1^ K^−1^. *κ*_ω_: lattice thermal conductivity from fully ab initio calculation; *κ*_transf_: approximated *κ*_ω_ with anharmonic force constants from Mg_2_Si; *κ*_forest_: *κ*_ω_ obtained random-forest regression; *κ*_anh_: *κ*_ω_ obtained with four exact anharmonic force constants and a linear model for the rest; *κ*_L_: lattice thermal conductivity form fully ab initio calculation in [76].

Materials	*κ*_ω_	*κ*_transf_	*κ*_forest_	*κ*_anh_	*κ*_L_
TiNiSn	17.9	57.1	20.3	16.8	15.4
ZrNiSn	19.6	73.3	20.7	17.5	13.3
HfNiSn	-	75.4	22.1	19.5	15.8
